# A conceptual framework for characterising lifecourse determinants of multiple long-term condition multimorbidity

**DOI:** 10.1177/26335565231193951

**Published:** 2023-09-03

**Authors:** Sebastian Stannard, Ann Berrington, Shantini Paranjothy, Rhiannon Owen, Simon Fraser, Rebecca Hoyle, Michael Boniface, Becky Wilkinson, Ashley Akbari, Sophia Batchelor, William Jones, Mark Ashworth, Jack Welch, Frances S Mair, Nisreen A Alwan

**Affiliations:** 1 School of Primary Care, Population Sciences and Medical Education, Faculty of Medicine, 12211 Southampton General Hospital, Southampton, UK; 2 Department of Social Statistics and Demography, 7423 University of Southampton, Southampton, UK; 3 School of Medicine, Medical Sciences and Nutrition, 1019 University of Aberdeen, Aberdeen, UK; 4 Population Data Science, Faculty of Medicine, Health and Life Science, Medical School, 7759 Swansea University, Swansea, UK; 5 School of Mathematical Sciences, 7423 University of Southampton, Southampton, UK; 6 School of Electronics and Computer Science, 7423 University of Southampton, Southampton, UK; 7 Public Health, 15577 Southampton City Council, Southampton, UK; 8 522468 The Alan Turing Institute, London, UK; 9 Patient and Public Involvement, 7425 University Hospital Southampton NHS Foundation Trust, Southampton, UK; 10 School of Life Course and Population Sciences, 4616 King’s College London, London, UK; 11 2996 Public Contributor on MELD-B, Southampton, UK; 12 General Practice & Primary Care, School of Health and Wellbeing, 3526 University of Glasgow, Glasgow, UK; 13 NIHR Southampton Biomedical Research Centre, 574429 University of Southampton; 7425 University Hospital Southampton NHS Foundation Trust, Southampton, UK; 14 NIHR Applied Research Collaboration Wessex, Southampton, UK

**Keywords:** Multimorbidity, early life, childhood, risk factors, conceptual framework

## Abstract

**Objective:**

Social, biological and environmental factors in early-life, defined as the period from preconception until age 18, play a role in shaping the risk of multiple long-term condition multimorbidity. However, there is a need to conceptualise these early-life factors, how they relate to each other, and provide conceptual framing for future research on aetiology and modelling prevention scenarios of multimorbidity. We develop a conceptual framework to characterise the population-level domains of early-life determinants of future multimorbidity.

**Method:**

This work was conducted as part of the Multidisciplinary Ecosystem to study Lifecourse Determinants and Prevention of Early-onset Burdensome Multimorbidity (MELD-B) study. The conceptualisation of multimorbidity lifecourse determinant domains was shaped by a review of existing research evidence and policy, and co-produced with public involvement via two workshops.

**Results:**

Early-life risk factors incorporate personal, social, economic, behavioural and environmental factors, and the key domains discussed in research evidence, policy, and with public contributors included adverse childhood experiences, socioeconomics, the social and physical environment, and education. Policy recommendations more often focused on individual-level factors as opposed to the wider determinants of health discussed within the research evidence. Some domains highlighted through our co-production process with public contributors, such as religion and spirituality, health screening and check-ups, and diet, were not adequately considered within the research evidence or policy.

**Conclusions:**

This co-produced conceptualisation can inform research directions using primary and secondary data to investigate the early-life characteristics of population groups at risk of future multimorbidity, as well as policy directions to target public health prevention scenarios of early-onset multimorbidity.

## Introduction

Multiple Long-Term Conditions Multimorbidity (MLTC-M) is defined as the co-existence of two or more chronic conditions, each one of which is either: a physical non-communicable disease of long duration, a mental health condition of long duration, or an infectious disease of long duration.^1^ The National Institute for Health Research (NIHR) supports the use of the term MLTC-M given they recognise that the public and patients do not like the term multimorbidity, they do not consider themselves to be multi-morbid, and it is the preferred nomenclature term for many people with MLTC-M and their families.^1^

MLTC-M occurs earlier in the lifecourse among people from more disadvantaged backgrounds, and among people from certain ethnic groups.^2,3^ While often considered a condition of older age, most people living with MLTC-M are under 65.^2^ Wider determinants such as family structure, education, housing, neighbourhood and work influence physical and mental health through multiple mechanistic pathways across the lifecourse.^4^ A substantial body of evidence points to the very early part of life as being crucial in determining health in childhood and in later years. Developmental Origins of Health and Disease (DOHaD) has become an established research field linking the aetiology of disease in adulthood with environmental exposures in utero and early-life.^5^ Pre-conception and pregnancy are important periods, and the concept of ‘fetal programming’ has emerged whereby a stimulus or insult during those periods can permanently affect the structure, physiology and metabolic system of offspring.^6–9^ Epigenetics is a biological pathway underlying DOHaD, where permanent effects of transient environmental influences alter epigenetic gene regulation.^10,11^ Despite this, there has been less evidence on the influence of wider social, biological and environmental factors across the whole of early-life, defined as the period from pre-conception until age 18, on the acquisition, age of onset, progression and combinations of Long-Term Conditions (LTCs).

We aim to conceptualise the domains of early-life determinants for population groups at risk of MLTC-M by scoping the research evidence and relevant policy documents, expert consensus, and refining this conceptualisation through a co-production process with public contributors. We believe that domain conceptualisation of these factors can make it easier to analyse big data in relation to early life risk of MLTC-M and to engage policy makers and practitioners in acting on the wider determinants of MLTC-M. This characterisation can also provide conceptual framing for future research on the aetiology and modelling of public health prevention scenarios of MLTC-M. This work is conducted as part of the Multidisciplinary Ecosystem to study Lifecourse Determinants and Prevention of Early-onset Burdensome Multimorbidity (MELD-B) project,^12^ which aims to use an artificial intelligence enhanced analysis of birth cohort data and Electronic Healthcare Record (EHR) data sources to identify lifecourse time points and targets for the prevention of early-onset, burdensome MLTC-M.

Risk factors for disease are interlinked and can cluster together, so separating them into independent domains is challenging. However, it is important to research clusters of risk not just individual risk factors, which is the traditional epidemiological approach. It is helpful that early-life risk factors are structured into domains as this will provide the conceptual framing to allow a shift in our approach to risk. This shift in approach will help to identify populations at higher risk and understand how to address the clustering of vulnerabilities to future ill health.

In phase one of the work, we scope the literature, as summarised in the next section. The subsequent section describes phase two, where we detail how these domains were refined with the guidance of public contributors. The final section briefly describes the next relevant steps in the MELD-B project.

## Phase 1: conceptualised domains of early-life determinants of future MLTC-M

We aimed to produce a conceptualisation of the grouping of potential early life determinants of MLTC-M. We utilised the scientific expertise within the MELD-B Consortium as a starting point. We then considered relevant literature following a multi-stage approach. Firstly, we searched for systematic reviews of early life determinants of multimorbidity. However, given the limited results, we expanded this search to include systematic reviews of determinants of multimorbidity or comorbidity from across the life course. Key concepts and terms underpinning the search encompassed ‘multimorbidity’, ‘comorbidity’, ‘long-term conditions’, ‘childhood’, ‘early-life’, ‘determinants’ and ‘risk factors’, and databases included PubMed and Google Scholar. Additionally, we reviewed relevant policy documents from across England and Wales from 2010 onwards that related to children, particularly in early-life. Our aim was not to capture all the evidence or conduct a systematic review of the subject but to inform the conceptualisation of our domains and build on expert opinion within our MELD-B Consortium. We therefore scoped the evidence for examples of these domains.

**Figure 1.**
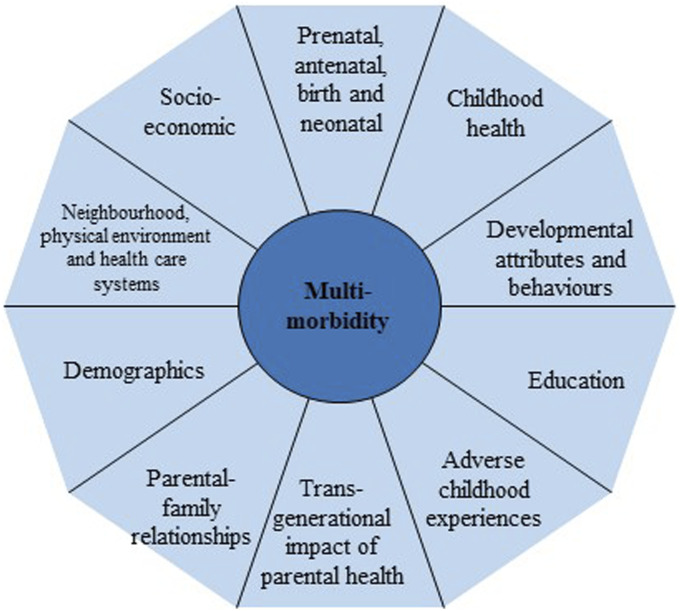
Conceptual early-life domains based on research evidence and policy.

Based on this scoping of the relevant scientific evidence and policy documents and MELD-B consortium expertise, we initially classified early-life determinants into 10 domains as described in Figure 1. Our classifications were loosely based on the Dalgren and Whitehead model of health determinants ^13^ and the Institute of Medicine multilevel approach to epidemiology.^14^ We provide a short summary of these domains below. We acknowledge overlaps between domains and that some variables can be recorded/collected across multiple domains. We also do not report all potential variables within each domain but provide a few examples given that the remit of this paper is the conceptualisation of these domains rather than a data landscape audit.

*Domain 1: Prenatal, antenatal, neonatal and birth (from conception to the first month of life)* focuses on the period from preconception to the onset of labour, the circumstances and outcomes surrounding a birth, and the period immediately following birth. Factors include birth size/weight, being born full term or prematurely, maternal diet and nutrition during and before pregnancy, smoking, alcohol consumption and drug misuse, maternal stress, breastfeeding, immunisation, safe pregnancy and birth surroundings.

Preconception is a crucial period that shapes risk of later adverse health outcomes for mother and child.^15,16^ Research has found that earlier gestational age at birth is associated with an increased risk of multimorbidity in adolescence (age 10-17 years) and early adulthood (age 18-30 years).^17^ Maternal smoking, drinking, drug use and obesity are also well-known modifiable risk factors for adverse child outcomes such as intrauterine growth restriction, obesity, cardio-metabolic, respiratory and cognitive-related health outcomes.^17–20^ The 2013 Welsh Government’s ‘Building a Brighter Future: Early Years and Childcare Plan’ documented the significance of the period from pre-birth to the end of the foundation phase (age seven years), where the fundamentals for good health are set.^21^ Marmot’s 2010 and 2020 reviews on child inequalities recommended pre- and postnatal support and routine support to families, including improved parenting programmes.^4,22^

*Domain 2: Adverse childhood experiences (ACE)* describe a range of negative experiences or events suffered in childhood and incorporating abuse, neglect or other traumatic experiences such as physical abuse, emotional abuse, sexual abuse, domestic violence, parental substance abuse, parental death, parental separation, and parental incarceration.^23^ Central to ACEs is the notion of ‘chain of risk’,^24^ where early-life stressors shape biological, physiological and psychological systems, setting children on developmental trajectories that may result in repeated exposure and heightened vulnerability to accumulating stressors across the lifecourse.^25^

Research has highlighted that parental death, exposure to violence, childhood abuse, and other childhood trauma to be important determinants of psychiatric comorbidity.^26^ Child maltreatment (physical/sexual/emotional/neglect) are also associated with higher Long Term Conditions (LTC) counts in adulthood.^27^ The 2019 UK Health and Social Care Select Committee report recognised the importance of ACEs (such as parental conflict, separation, and parental drug or alcohol problems) on children’s development, health, and life chances. The report highlighted that adversity during childhood can influence future parenting behaviour, resulting in cycles of disadvantage.^28^

*Domain 3: Child health* considers the health of a child from birth to age 18. Factors include genetics, health behaviours, and biomarkers. The domain covers both the mental and physical health of a child, parental health decisions such as immunisations and health care visits and service access.

Physiological markers of multimorbidity across the life course include dehydroepiandrosterone sulphate (DHEAS), Interleukin 6 (IL-6), C-reactive protein (CRP), Lipoprotein (Lp), and Cystatin C (Cyst-C).^29^ Significant biomarkers of multimorbidity included serum, molecular, physiological (oral temperature, blood pressure, heart rate), body size (BMI), and brain functioning.^30^ Cerda et al.,^26^ found genetic determinants of psychiatric comorbidity to include 5HTTLPR, MAOA, and DRD1- DRD4. Poorer health in childhood such as asthma, hearing impairments, general health and low height have been found to be related to long-term health outcomes across the adult life course.^31^ Children who experience more illnesses at a young age have also been found to be more prone to developing multimorbidity in later life.^32^

The first 1000 days of life report concluded that child health is a determinant of future multimorbidity,^28^ whilst lower body mass index at age ten was associated with a reduced risk of early-onset multimorbidity.^33^ Good health factors (adequate nutrition, warmth, exercise, sleep, protection from infectious disease, and environmental hazards) were also important foundations for future health, highlighted by the 2022 Public Health Wales report.^34^

*Domain 4: Developmental attributes and health behaviour* focuses on the developmental markers of children and incorporates measures relating to cognition, coordination, personality and behaviour. This domain also incorporates health-related behaviours (for example, smoking, diet, physical activity, alcohol consumption, drug misuse and sleep patterns), and includes neurodevelopmental conditions that could affect behaviours such as ADHD and autism.

Important determinants of multimorbidity have been found to be characterised by risk factors that include psychological indicators such as thoughts, emotions, and behaviour,^35^ and higher cognitive ability aged ten is associated with reduced early-onset multimorbidity.^33^ Health behaviours have also been found to predict longitudinal multimorbidity outcomes,^36^ and physical activity in childhood has been found to be protective against multimorbidity at older ages.^37^ The Early Intervention Foundation argued that child behaviour (the ability to monitor and regulate own behaviour, attention and impulses) influenced health across the lifecourse.^38^

*Domain 5: Child education* relates to the process of learning, especially in educational settings, and the knowledge an individual gains from these educational institutions. Factors include formal educational grades, academic ability tests, and educational tests.

Pathirana and Jackson^39^ found levels of education and literacy to be important determinants of multimorbidity, and in the Australian Longitudinal Study on Women’s Health, lower educational attainment was associated with increased multimorbidity risk.^37^ Recommendations from the 2010 and 2020 Marmot Reviews aimed at ensuring children have the best start in life, included school readiness and attainment, and good quality early years education.^4,22^ Recommendations from the Royal Foundation of the Duke and Duchess of Cambridge included the importance of promoting education, and supported wide dissemination of evidence on the primacy of the early years.^38^ However, given research demonstrating that children with neurodevelopmental multimorbidity are at greater risk of poorer educational outcomes,^37^ it is important to note that the relationships between child education and multimorbidity may be bidirectional.

*Domain 6: Demographics* is an umbrella term that refers to factors that help describe the size, structure and distribution of populations, and incorporates factors such as age, ethnicity, sex, marital status, migration, income and education.

Variations in MLTC-M incidence by ethnicity and childhood disadvantage have been linked to adverse health in adulthood.^40–44^ Determinants of multimorbidity have also been characterised as either biophysiological or somatic risk factors that included demographic characteristics (age, sex, ethnicity), sociodemographic characteristics and social networks.^35^ Important area-level demographic determinants of multimorbidity include deprivation, household composition, social class, and household primary language.^33^ Multimorbidity patterns have also been found to differ according to migration status.^44^

*Domain 7: Transgenerational impact of parental health and behaviours* refers to factors that can be transmitted across generations. Examples include parental health behaviours (diet, smoking, alcohol consumption, substance misuse, and exercise), and wider health literacy (self-efficacy, knowledge, and use of health information).^45–47^

The 2022 Institute for Fiscal Study Deaton Review recognised that inequalities in health begin at home, with particular emphasis on economic circumstances, parental mental wellbeing and parenting behaviours.^48^ The review acknowledged that children’s health is influenced, to varying degrees, by a broad range of proximal factors, such as genetics, the family environment and major life events, as well as distal factors, including neighbourhood characteristics, climate, and culture.^49^ The review discussed the critical role of the primary caregivers who, through the emotional environments they are able to provide, exert a profound and lasting influence on children’s emotional regulation and subsequent behaviour, interactions and relationships; and who have a profound influence on cognitive development.

*Domain 8: Socioeconomic factors* is an umbrella term that refers to factors concerned with the interaction of social and economic issues and incorporates a combination of variables relating to education, income, employment and occupation.

Socioeconomic disadvantage is key in shaping developmental life experiences.^50^ Analyses in the Hertfordshire cohort study showed paternal social class was associated with future multimorbidity.^32^ In the Aberdeen Children of the 1950s (ACONF) cohort, lower father’s social class at birth was associated with early-onset multimorbidity.^51^ In the 1970 British Cohort Study (BCS70), those with fathers from unskilled occupational groups (vs. professional) at birth had a 43% increased risk of early-onset multimorbidity.^33^ Data from the English Longitudinal Study of Ageing showed that those with the lowest wealth had 47% higher odds of basic multimorbidity (95% C.I. 1.34-1.61) and 73% higher odds of complex multimorbidity (95% C.I. 1.52-1.96) compared to those with the highest wealth.^52^ There were also associations between household income, low education, occupational status and multimorbidity.^53^ The 2022 Public Health Wales report highlights that families require sufficient income to provide a clean, warm home, nutritious food, clothing, activities and equipment. Financial difficulties can increase the risk of parental stress and poor mental health, leading to poorer outcomes for children.^34^

*Domain 9:* The *parental and family environment* will be the main source of development and stimulation until nursery/school. This domain incorporates parental-child interactions and the interaction between children and the primary care giver, parenting styles, parental beliefs, attitudes and discipline, and wider family factors such as kin networks.

Research has suggested that the impact of parents may never be greater than during the earliest years of life, when nearly all of their experiences are created and shaped by parents and the circumstances within their family environment.^48^ The 2013 Welsh Government’s ‘Building a Brighter Future: Early Years and Childcare Plan’ recognised that developmental factors, including parental factors (parental health, lifestyle and behaviours, parental interest in the child and the quality of the child’s relationships) are important predictors of child health.^21^ The 2022 Public Health Wales report highlights that the relationship between the parent and child, between the child’s parents, and the family’s relationships with their wider family, are key components influencing children’s wellbeing and development.^34^ Parenting and family support, parental warmth, parental lack of aversive behaviour and disciplinary skills are also important determinants of psychiatric comorbidity.^45^

*Domain 10: Neighbourhood, the physical environmental and health care systems.* This domain incorporates wider external factors relating to neighbourhoods and the physical environments such as trust in neighbourhood, condition of neighbourhood, rural/urban neighbourhood, access to parks and outdoor spaces, access to local amenities (library/schools/shops/post office) and access to medical services (pharmacy/GP surgery/hospital). This domain therefore partly embraces the concept of social capital focusing on the social relationships and network of relationships amongst people.^54^

Cezard et al.,^36^ Cerda et al.,^26^ and Ingram et al.,^44^ all conclude that wider determinants of multimorbidity comprise area-level deprivation, rurality, service access, peer group association, trust in neighbourhood and service use. Geographic indices of deprivation, such as the Carstairs and Townsend Index, also predict multimorbidity.^55,56^ Levels of crime and violence in an area, and areas of high economic deprivation and low socioeconomic status have also been found to be associated with multimorbidity.^44^ Services access and availability, including primary care, health visiting, midwifery, housing, mental health services, childcare, education, social services, family support, and sometimes probation and prison services, are key factors in improving children’s health, development and life chances.^41^ Communities and the local environments can influence parents’ own health and wellbeing, their parenting norms and behaviours and their ability to access support, whilst the social infrastructure of neighbourhoods can reduce social isolation and promote social cohesion.^34^

## Phase 2: Refining the domains through a co-production process with public contributors

The second stage of our conceptualisation involved refining our domains with public contributors. This phase followed NIHR guidance suggesting that PPI involvement is done with or by patients and the public rather than to, about, or for.^57^ PPI is recognised as a method to fill in the gaps between research and lived experience,^58^ and PPI involvement in our research allowed us to gain insights into factors relevant to both young adults and people with lived experience of MLTC-M, to understand domains that may have been overlooked in research evidence and policy, and identify factors that are important to patients and the public.

## Patient and Public Recruitment

We sought the views of young adults (age 18-30) regardless of MLTC-M status and older adults (age 40-65) living with self-reported MLTC-M (either mental or physical health conditions or both). Although the latter were experiencing the outcome of interest, we felt it was important to understand younger people’s more recent lived experience of early-life. We also felt it important to capture a young cohort perspective of health since the overall aim of the MELD-B project is to gain an understanding of health across the life course. However, we decided not to hold workshops with children under the age of 18 given the complex and sensitive nature of the topic, we also wanted public contributors to be able to reflect on the whole of childhood (up to the age of 18).

A call for public contributors living in the UK was circulated via social media (Facebook, Instagram and Twitter), and potential participants who fulfilled the selection criteria were encouraged to contact the research team by email or via an online form. A screening questionnaire was administered to check eligibility and ensure that the public contributors were selected from a range of demographic backgrounds (age, gender, ethnicity and geographical location). We included 8 PPI members in the young adult group and 12 PPI members in the older adult group. Across the two groups we had 8 men, 10 women and 2 individuals who identified as either transgender or non-binary. Across both workshops we had an ethnically diverse group of individuals, with public contributors identifying as either Black, African, Hispanic, Caribbean, Mixed Race or Asian.

We held two online 1.5-hour workshops. The workshops explored PPI members’ awareness of factors and events in childhood that they felt may influence health in midlife. PPI members were encouraged to list and discuss the top 5 factors in childhood for future health. Example questions included: ‘Do you feel that factors or events in childhood could influence a person’s health at midlife, and if so, why?’; ‘What are the top 5 factors or events in childhood that you feel may influence a person’s health?’; ‘Why did you choose these 5 factors?’ We presented three figures to help stimulate discussions (these are included in Supplementary figures 1-3), and we used ‘Jamboard’^59^ an online interactive whiteboard, to allow people to respond anonymously to each question via virtual ‘sticky notes’. We did not explicitly share the findings from the scoping review with the public contributors to avoid biasing their opinions, but discussion that centred on the individual domains was encouraged. PPI members who completed the workshops were offered ‘e-shopping’ vouchers. The value of the voucher (£40) was in line with the NIHR rate for PPI involvement.^60^

## Ethics Approvals

The study is conducted in accordance with the UK Policy Framework for Health and Social Care Research. Ethics approval has been obtained from the University of Southampton Faculty of Medicine Ethics committee (ERGO II Reference 66810).

## The PPI workshops

### Workshop 1 – Public contributors aged 18-30 years

**Figure 2.**
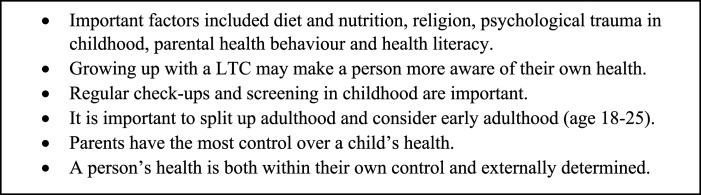
The key discussion points from workshop 1 (younger group).

Key points from the first workshop held with the younger group (age 18-30) are highlighted in Figure 2. The participants in this workshop were not selected according to MLTC-M status, although we did have a number of participants with LTCs. The group discussed both the impact of poor nutrition and the role of consuming certain foods in childhood for predicting future health. They debated how children who live with a LTC or grow up in a family where a family member has a LTC may be more aware of their own health, and may be more likely to approach health services when appropriate. The group discussed how religion could influence both health behaviours and health decisions such as contraceptive use, alcohol consumption and diet. They discussed the importance of regular screening and regular check-ups in childhood. There was a debate as to whether health is within an individual’s control or externally determined. The group discussed how individuals have control over their day-to-day lives but suggested that there is a random element partly responsible for the development of a LTC. Finally, the group suggested the research team should consider distinguishing early adulthood (age 18-25) as a distinct phase of adulthood, given that this is a crucial period in the transition from childhood to adulthood, and where events during this period may have a particularly strong impact across the lifecourse.

### Workshop 2 – Public contributors over 40 years with MLTC-M

**Figure 3.**
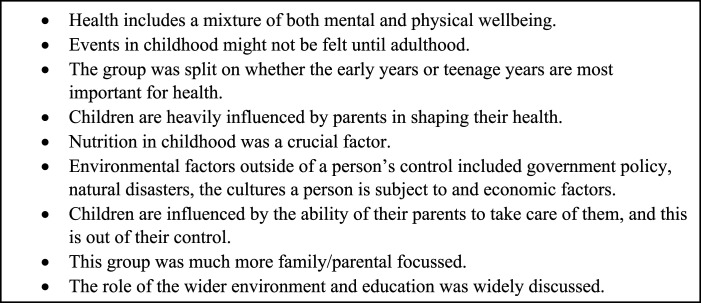
The key discussion points from workshop 2 (older group).

The second workshop was with an older age group (age 40-65), all with MLTC-M described as at least two LTCs. Key points from the second workshop are highlighted in Figure 3. The group discussed how the impact of events in childhood (such as child stress, parental smoking, environmental hazards, trauma, and injuries) might not be felt until adulthood, and that poor health may result from one big event or many smaller events that accumulate over time. The group discussed how health literacy and knowledge are passed down from parents to children and that children are influenced by the ability of their parents to take care of them. They discussed how it is not just education that is important for promoting good health, but rather the wider sources of context and knowledge people can gain information from about being healthy. There were mixed views around the age at which events might have the greatest impact on health. Some of the group argued that the early years (age 0-7) were most important given this is when there is most room for improvement and learning, where children are heavily influenced by their environment. However, others in the group argued that adolescence (age 9-15) is more important, given that this is when children develop the fastest and are more exposed to external factors. The group discussed how during the teenage years, teenagers want to test boundaries and learn for themselves, and therefore events during this period might have a larger impact. Consistent with the discussion in the younger PPI group, they suggested that whilst many factors are within an individual’s control, there are external factors which affect health (examples included government policy, natural disasters, wider culture, and economic factors).

## How the PPI workshops shaped the conceptual domains

The prenatal, antenatal, birth and neonatal period and developmental attributes were not discussed by either group of public contributors as being important to the development of long-term health. However, the public contributors supported our decision to group early-life domains for population groups at risk of future MLTC-M into domains that focused on demographic factors, adverse childhood experiences, socioeconomic conditions, physical environment and healthcare services, and neighbourhood characteristics. The PPI workshop discussions suggested that a number of the domains should be expanded further: childhood health should be expanded to include check-ups and screening, and parental-family relationships should be expanded to include a parent’s ability to care for a child. Other domains that required expanding included the intergenerational impact of parental health to include the intergenerational transmission of parental health behaviours and education to include health literacy. Following the PPI workshops it was clear that two further domains should be conceptualised:

*Domain 11: Health behaviours and diet.* This domain is conceptualised separately to others because the public contributors suggested creating a domain such that child health behaviours and diet are separated from child health and developmental attributes. This domain is therefore an expansion of the original domain 4 – ‘developmental attributes and behaviour’ and incorporate health-related behaviours (under the age of 18) such as smoking, diet, physical activity, alcohol consumption, drug misuse and sleep patterns. This domain also considers diet focusing on objective measures of both nutrition and diet, as opposed to anthropometric measurements.

*Domain 12: Religion, spirituality and wider culture.* The role of religion, spirituality and wider culture was discussed by public contributors. Since this area of research had not been highlighted in the scoping of research evidence or policy documents, we proposed this as an additional 12^th^ domain. This domain incorporates the role of religion, spirituality and wider cultural norms and attitudes on influencing health, health literacy health behaviours and health care decisions such as contraceptive use, fertility patterns, blood transfusions, medicines based on animal products, and vaccination hesitancy.

**Figure 4.**
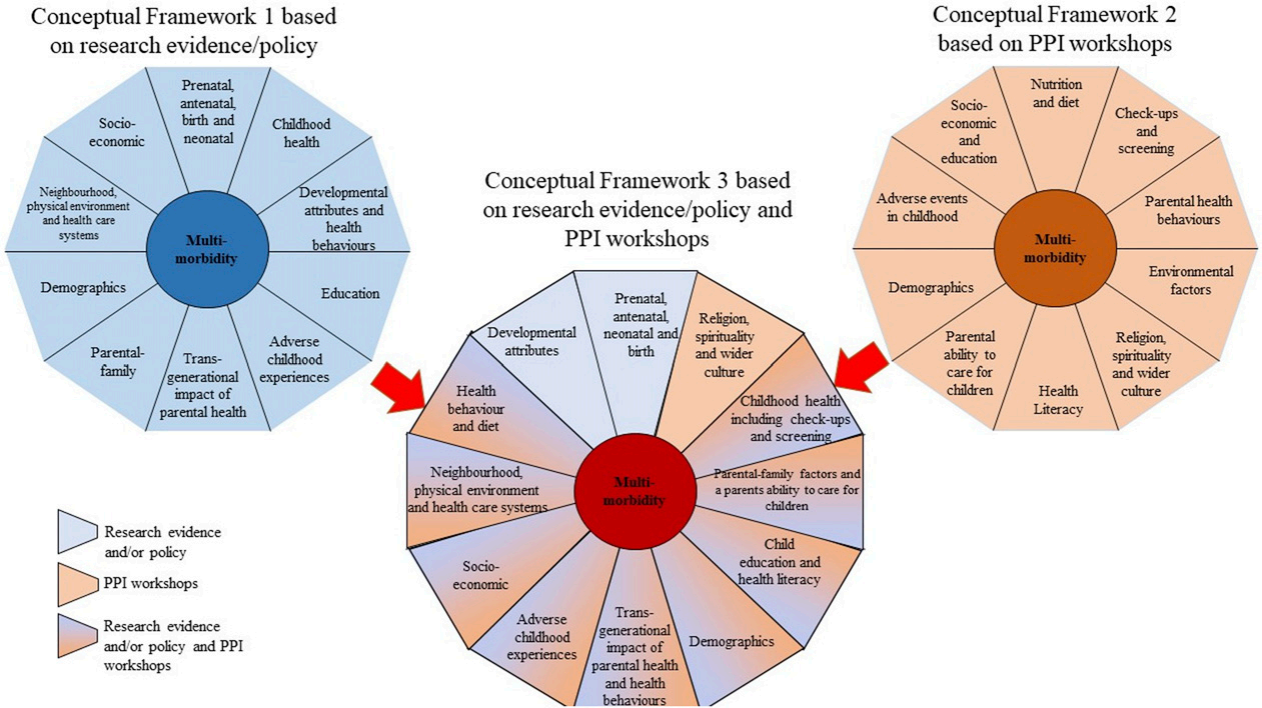
The development of the conceptual framework.

**Figure 5.**
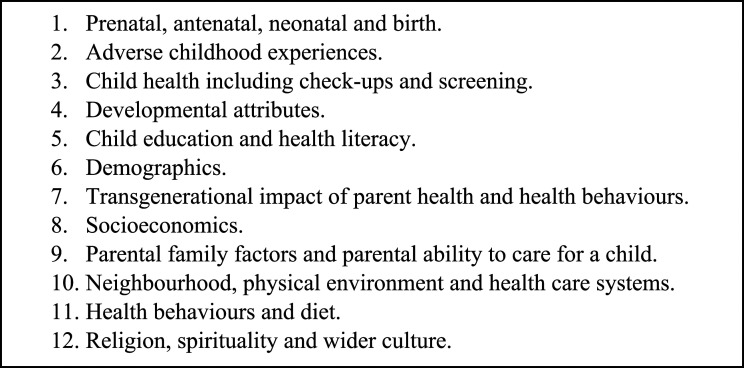
The 12 domains of early-life risk factors identified from the literature, policy and PPI contributions.

As shown in Figure 4, two conceptual frameworks were created; one conceptual framework identified domains of early-life risk factors from research evidence and policy, as discussed in phase 1 (Conceptual Framework 1). The second conceptual framework identified domains of early-life risk factors from the two PPI workshops, as discussed in phase 2 (Conceptual Framework 2). We mapped a final conceptual framework (Conceptual Framework 3) that combined conceptual frameworks 1 and 2, this was achieved by combining domains from the first two conceptual framework with considerable overlap, and retaining those domains that did not overlap as separate domains. This led us to conceptualise the final 12 early-life domains for population groups at risk of future MLTC-M early-life risk factors (Figure 5).

## Next steps in the Meld-B project

### Step 1. Mapping datasets onto the conceptual framework

This project's next step involves a data landscape audit and mapping the conceptualised domains to the available and relevant variables. This will be done using multiple data sources, including longitudinal birth cohort studies and routinely-collected EHR data sources, to ensure a supplementary range of early-life risk factors are considered. Our future modelling will build on Kuh and Ben-Shlomo's definition of life course epidemiology ‘the study of long-term effects on later health or disease risk of physical or social exposures during gestation, childhood, adolescence, young adulthood and later adult life’.^61^ Most significantly and outlined by Kuh and Ben-Shlomo, utilising a life course approach to epidemiology,^62^ our research will focus on early life risk factors whilst acknowledging the importance of conventional risk factors for MLTC-M (such as smoking, diet, physical exercise, socioeconomic status, ethnicity) across the life course.

Our MELD-B project will use the *Aberdeen Children of the 1950s (ACONF),* which includes children born in Aberdeen, Scotland between 1950 and 1956; in total there are 12,150 cohort members, and participants are linked to hospital and mental health admissions, maternity records, cancer registers, and death records.^63^ The *National Child Development Study (NCDS)*^64^ which has followed all children born in England, Scotland and Wales in one week in 1958, and the *1970 British Cohort Study (BCS70)*^65^ which has followed all children born in England, Scotland, Wales and Northern Ireland in one week in 1970 will also be used. Both the NCDS and BCS70 have collected information on social, economic, biological and environmental factors and can be linked to hospital episode statistics. Finally, MELD-B will access a range of routinely collected EHR data sources within the *Secure Anonymised Information Linkage (SAIL) Databank,* a privacy-protecting trusted research environment.^66^ SAIL contains linkable anonymised individual-level population-scale data, including primary and secondary care, health and social care, demographic, geographic, socioeconomic, birth and mortality data sources for the whole population of Wales.

### Step 2. Statistical analysis

The overarching aim is to identify the nature and optimal timing for potential interventions for the prevention of early-onset, burdensome MLTC-M and generate research evidence to inform public health initiatives on prevention of MLTC-M. We will draw on methods from statistics, mathematical modelling, and machine learning to summarise early-life risk factor clusters and their associations with MLTC-M.

The data sources above will be curated in order to provide an accessible research ready data asset (RRDA) to further understand the domains conceptualised in this paper and the early-life determinants of MLTC-M through the use of epidemiological and machine learning approaches. Our collaborative team will evaluate statistical, mathematical modelling and machine learning methods to address the following broad research objectives:

• To identify and characterise clusters of early-life exposures and characterise population groups at risk of future MLTC-M in early-life (prebirth-18 years).
• To identify critical time points and key lifecourse targets for MLTC-M prevention and model counterfactual prevention scenarios acting on combined risk factors at the specified timepoints (prebirth-18 years).
• To investigate the influence of sentinel conditions and sequence of accrual of wider determinants, conditions and burdensome factors in the development of early-onset, burdensome MLTC-M clusters.
• To compare machine learning and causal inference modelling for identifying potential early-life (0-18) ‘preventable moments’ in life trajectories and exploring alternative trajectories based on models of policies/strategies/interventions and outcomes.

## Discussion

While much research has considered wider societal and economic factors on MLTC-M, the full picture of the influence of early-life risk factors has not been conceptualised. This paper has helped to address this issue by conceptualising domains that incorporate a wide range of personal, social, economic and environmental factors in early-life that may affect people’s risk of future MLTC-M. We identified 12 domains of early-life risk factors from research evidence and policy, and with the support of public contributors. These domains could be used to inform future MLTC-M research, especially where the focus is early-life. Some domains highlighted through our co-production process with public contributors, such as religion and spirituality, health screening and check-ups, and objective measures of diet, as opposed to anthropometric measurements often used as indicators of nutritional or dietary status, were not adequately considered when reviewing the research evidence or relevant policy documents. These require further consideration of data availability to investigate them in the context of MLTC-M prevention.

We believe this domain conceptualisation will make it easier to engage policy makers and practitioners in acting on the wider determinants of MLTC-M. The benefit of conceptualised models is that they can help to convey ideas to diverse audiences, provide improved guidance for prevention and intervention efforts, and provide visual representation of specific research questions as described by Brady et al.^67^

Our conceptualisation could be used to depict a set of risk and protective factors that may be associated with the development of MLTC-M across the life course and provide a better understanding of the wide range of early life factors that may influence health. In turn, a better understanding of what influences health can lead to the development of more effective public health interventions and policies, as well as more efficient use of public health resources. Future research can use our framework to explore in more detail and understand the relationship between these co-produced domains and MLTC-M risk. This framework can also help move beyond causal inference analyses that consider the impact of one early-life determinant on later multimorbidity risk to the consideration of how the combined impact of multiple early-life characteristics at different lifecourse timepoints can be facilitated or disrupted.

The concept of developing individual resilience against the adverse health outcomes of multimorbidity is a field of growing interest and research.^68,69^ This conceptual framing of early life risk of multimorbidity can be regarded as a step towards conceptualising population-level resilience to prevent or delay the burden of multimorbidity on society. This conceptualisation also supports wider ongoing debates within the UK healthcare system about shifting towards a more preventive model of health. The Department of Health and Social Care 2021 policy paper on transforming the public health system^70^ highlighted the need to focus on prevention and the wider determinants of health, and the 2018 paper on the Public Health Priorities in Scotland^71^ included the need to invest early in young people's future as the best form of prevention. We therefore see our conceptualisation as an extension to these debates as we conceptualised domains for prevention of early onset MLTC-M.

Our conceptual model resonates with emerging literature on this research topic. Henchoz et al,^72^ discussed the importance of many factors we have highlighted (including premature birth, food restriction, child labour, family economic environment, serious illness/accident, and stressful life events), but their research did not derive these factors into conceptual domains. Emergent research has also considered the role of individual domains we consider, such as education,^73^ child adversity (child abuse and neglect, negative caregiver characteristics, and low socioeconomic status),^74^ early life deprivation,^75^ and socioeconomic conditions.^76^ However, our model goes beyond these studies as we conceptualise the full picture of influence of early-life risk factors.

## Strengths and limitations

Our call for public contributors to discuss early life determinants of MLTC-M received a good response from potential public contributors. Advertising on social media is both a strength and a limitation in the sense that it can have potentially much wider reach, but may exclude people with potentially different characteristics and views who do not routinely use social media. Most of those who participated in the workshops had no past PPI experience, and it was important to offer them clear information about the process and the support available. Although conducting the workshops online meant we were able to attract people from a wider geographical location, a limitation with the online workshops was that it was harder to stimulate discussions with some public contributors who were more reluctant to speak.

It was not our intention to conduct a systematic review of the subject but rather our aim was to inform the initial classification of the conceptual domains. However, it should be noted that not undertaking a systematic review is a limitation. Given the time and resources needed to conduct a systematic review it was not possible within the scope of the MELD-B project. However, a full systematic review (and meta-analysis if applicable) into the early-life determinants of MLTC-M could be an important next step for future research.

## Conclusions

Innovative conceptual modelling such as the model presented here, can help to challenge existing understanding of the aetiology of multimorbidity, develop new ideas and solutions, and facilitate improvements in the quality of clinical and public health responses to MLTC-M. We recommend a number of important next steps that include a data landscape audit, mapping the conceptualised domains to the available and relevant variables, and statistical analysis to better identify lifecourse time points and targets for the prevention of early-onset, burdensome MLTC-M. A strength of the MELD-B study is that these next steps will be achieved using a multidisciplinary approach, drawing expertise from a wide range of disciplines not always affiliated to health-related studies. These next steps will also be achieved with continuing PPI involvement, particularly when considering potential interventions and to ensure that the modelling frameworks used reflect the experienced disease pathways. This work will help support research communities and healthcare providers to aid prevention, healthcare planning and the management of individuals at risk of MLTC-M.

## Supplemental Material

Supplemental Material - A conceptual framework for characterising lifecourse determinants of multiple long-term condition multimorbidity Click here for additional data file. Supplemental Material for A conceptual framework for characterising lifecourse determinants of multiple long-term condition multimorbidity by Sebastian Stannard, Ann Berrington, Shantini Paranjothy, Rhiannon Owen, Simon Fraser, Rebecca Hoyle, Michael Boniface, Becky Wilkinson, Ashley Akbari, Sophia Batchelor, William Jones, Mark Ashworth, Jack Welch, Frances S Mair and Nisreen A Alwan in Journal of Multimorbidity and Comorbidity
