# Supplementary material for: A conceptual framework for characterising lifecourse determinants of multiple long-term condition multimorbidity
Source: J Multimorb Comorb. 2023 Sep 3;13:26335565231193951. doi: 10.1177/26335565231193951 (PMC10478563; doi:10.1177/26335565231193951)

Supplementary Figure 1. Life stages figure to help stimulate discussions with public contributors

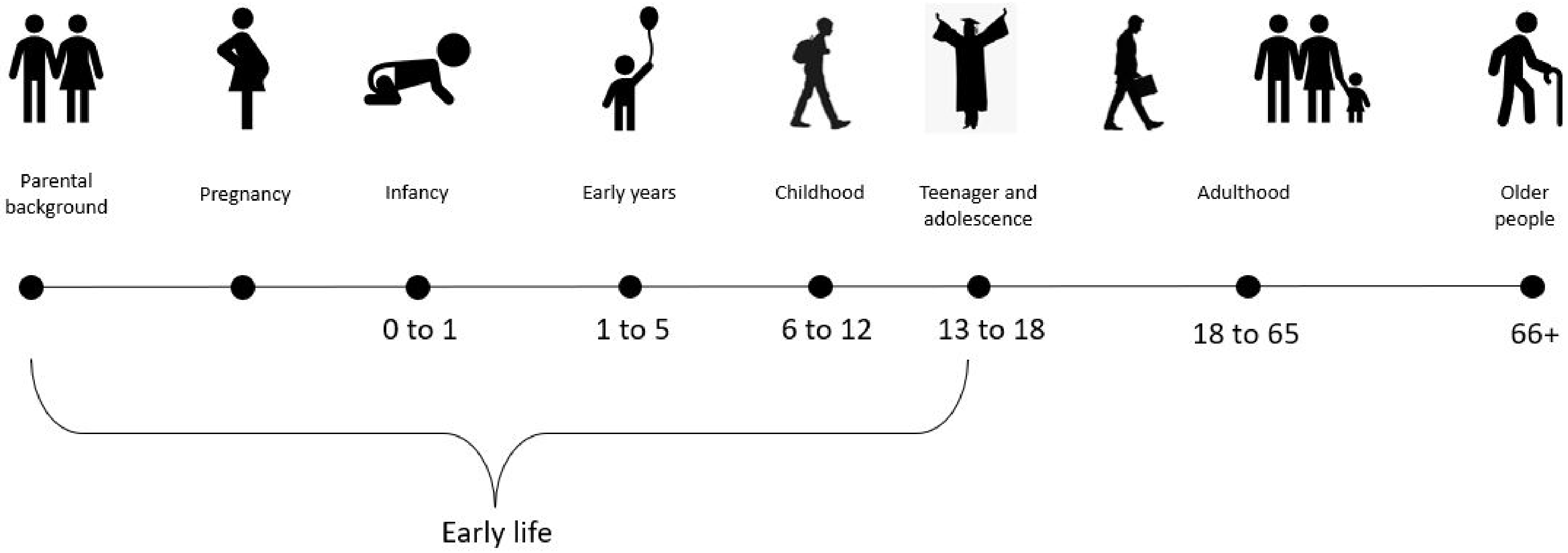

Supplementary Figure 2. Influences on multimorbidity across the lifecourse

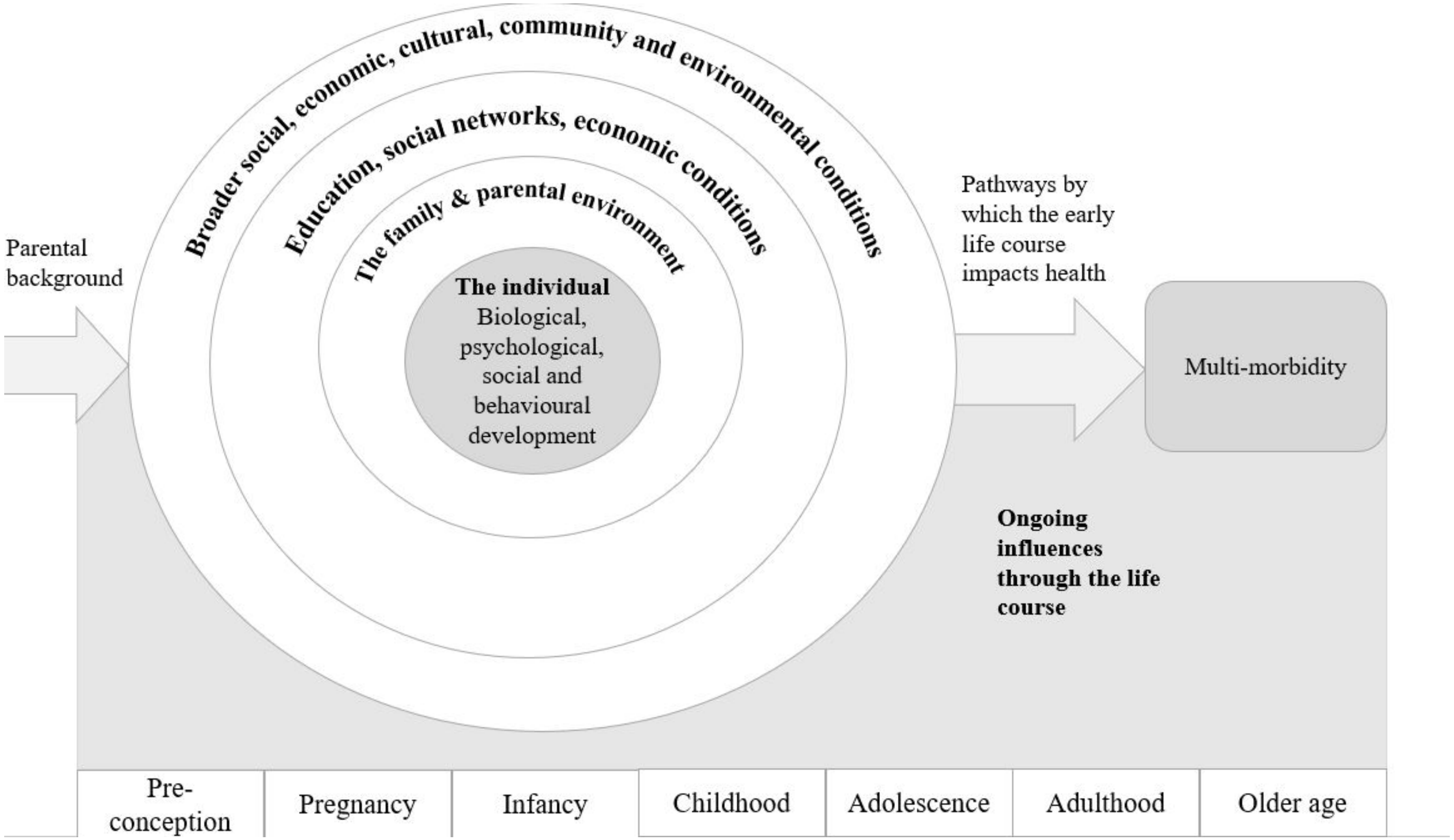

Supplementary Figure 3. Influences on multimorbidity in childhood

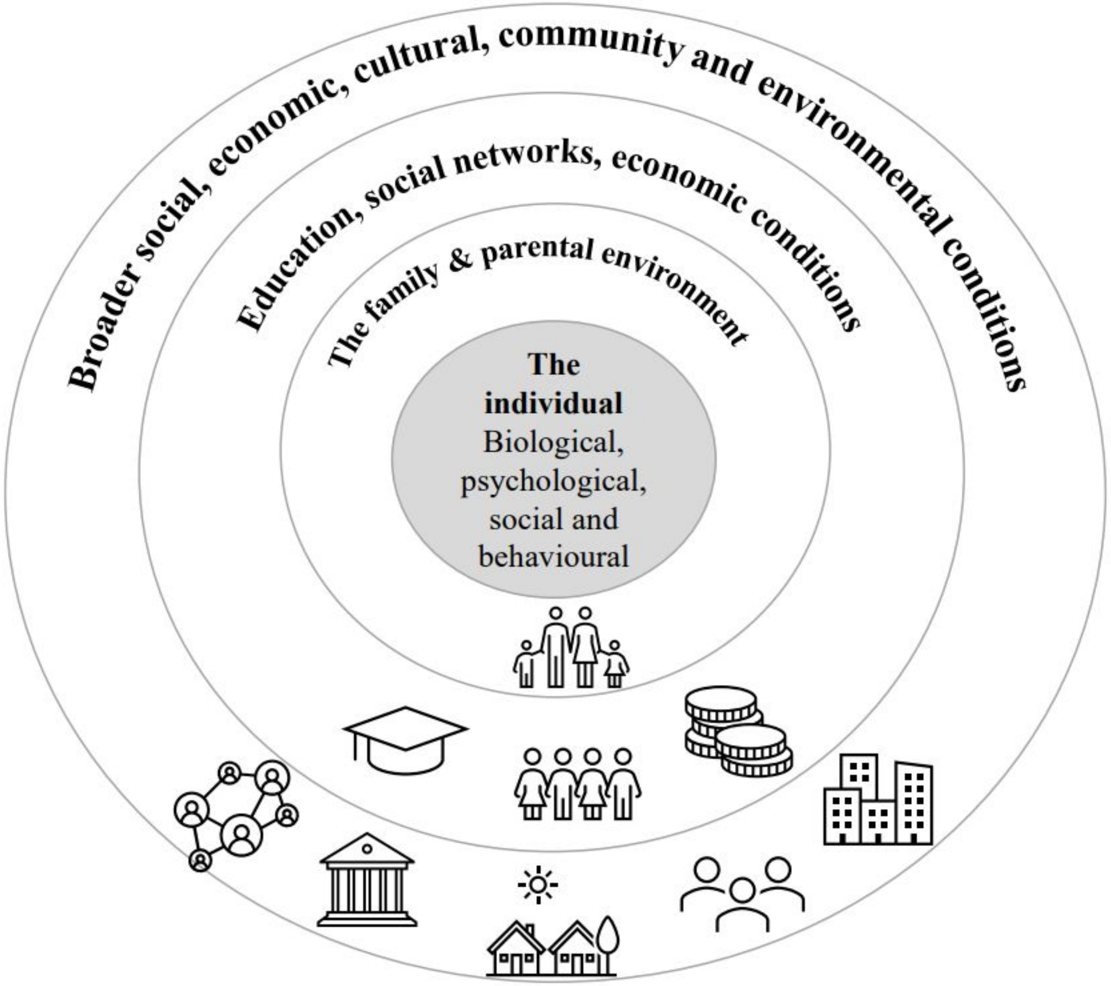

Supplement: Supplemental Material - A conceptual framework for characterising lifecourse determinants of multiple long-term condition multimorbidity [file sj-pdf-1-cob-10.1177_26335565231193951.pdf]
